# The health response to the Rohingya refugee crisis post August 2017: Reflections from two years of health sector coordination in Cox’s Bazar, Bangladesh

**DOI:** 10.1371/journal.pone.0253013

**Published:** 2021-06-11

**Authors:** Rosanna Jeffries, Hassan Abdi, Mohammad Ali, Abu Toha Md Rezuanul Haque Bhuiyan, Mohamed El Shazly, Sandra Harlass, Asm Ishtiak, Md Khadimul Anam Mazhar, Mukeshkumar Prajapati, Qing Yuan Pang, Balwinder Singh, Francis Tabu, Amrish Baidjoe

**Affiliations:** 1 World Health Organisation, Cox’s Bazar, Bangladesh; 2 United Nations Population Fund, Cox’s Bazar, Bangladesh; 3 Ministry of Health and Family Welfare Coordination Center, Cox’s Bazar, Bangladesh; 4 Refugee Relief and Repatriation Commissioner’s (RRRC) Office, Cox’s Bazar, Bangladesh; 5 United Nations High Commissioner for Refugees, Cox’s Bazar, Bangladesh; 6 International Organisation for Migration Cox’s Bazar, Bangladesh; 7 Department of Infectious Disease Epidemiology, London School of Hygiene and Tropical Medicine, London, United Kingdom; 8 World Health Organisation, Regional Office of South East Asia, Delhi, India; Jhpiego, UNITED STATES

## Abstract

On August 25 2017, an unprecedented influx of Rohingya refugees began from Rakhine State in Myanmar into Bangladesh’s district of Cox’s Bazar. The scale and acuteness of this humanitarian crisis was unprecedented and unique globally, requiring strong coordination of a multitude of actors. This paper reflects on the health sector coordination from August 2017 to October 2019, focusing on selected achievements and persisting challenges of the health sector strategic advisory group (HSSAG), and the health sector working groups including epidemiology and case management, sexual and reproductive health, community health, mental health and psychosocial support, and emergency preparedness. In the early days of the response, minimum service standards for primary health care were established, a fundamental initial step which enabled the standardization of services based on critical needs. Similarly, establishing standards for community health outreach was the backbone for capitalizing on this important health workforce. Novel approaches were adopted for infectious disease responses for acute watery diarrhoea and varicella, drawing on inter-sectoral collaborations. Sexual and reproductive health services were prioritized from the initial onset of the crisis and improvements in skilled delivery attendance, gender-based violence services, abortion care and family planning were recorded. Mental health service provision was strengthened through community-based approaches although integration of mental health programmes into primary health care has been limited by availability of specialist psychiatrists. Strong, collaborative and legitimate leadership by the health sector strategic advisory group, drawing on inter-sectoral collaborations and the technical expertise of the different technical working groups, were critical in the response and proved effective, despite the remaining challenges to be addressed. Anticipated reductions in funding as the crisis moves into protracted status threatens the achievements of the health sector in provision of health services to the Rohingya refugees.

## Introduction

From 25^th^ August 2017, the largest and fastest influx of Rohingya refugees fled from Rakhine State in Myanmar to Bangladesh’s district of Cox’s Bazar. As of October 2019, an estimated 911,566 Rohingya refugees resided in Cox’s Bazar district, of which 905,754 within 34 refugee camps [1], including Kutupalong which is the largest single refugee camp in the world by population size [2]. The camps span an area of 16.7 km^2^ with an average population density of 50,299 persons per km^2^ (compared to 44,500 persons per km^2^ in Dhaka Bangladesh, considered by some measures as the city with the highest population density globally) [3]. While findings from a December 2018 annual representative survey showed that crude mortality rates among the refugees were below WHO emergency threshold of 1 death/10,000 persons/day [4], the physical and mental health needs of this population group are substantial and varied, ranging from chronic illnesses to infectious diseases, injuries and physical disabilities, mild to severe mental conditions, sexual and reproductive health concerns, and emergency care. This reflects not only the physical and emotional impact of the 2017 violence which led the Rohingya to flee from their homes, but also a long-standing history of marginalisation of the Rohingya in Myanmar. The scale of health needs, compounded by Cox’s Bazar district’s predisposition to seasonal risk factors related to monsoons and cyclones [5], make this a truly unique crisis requiring a robust response to manage evolving needs, including during the transition from acute to protracted phase. This paper reflects on the health sector response from August 2017 to October 2019, describing some of its unique characteristics, successes and challenges, to draw lessons learned which may be useful in this and other humanitarian crises.

The health sector in Cox’s Bazar, which is equivalent to the “cluster” in other humanitarian settings, is coordinated under the leadership of the Ministry of Health’s Civil Surgeon’s Office of Cox’s Bazar, the Ministry of Health and Family Welfare Coordination Centre and the World Health Organization (WHO). Two-years into the crisis, the health sector coordination group comprised more than 100 partners and there were more than 200 health facilities in the refugee camps, covering primary, secondary, and specialized health services. Although the security situation fortunately remained stable throughout, which helped minimise direct threats to service delivery, strong coordination and collaboration were nevertheless essential for establishing and maintaining an effective, efficient and acceptable technical response of this scale, complexity and volatility.

## Health sector strategic advisory group

Since November 2017, the health sector has been led by a Health Sector Strategic Advisory Group (HSSAG), constituting representatives from government, the UN bodies and selected national and international NGOs. This HSSAG, which was established very rapidly, became stronger and more decisive with increasingly prominent leadership from both the government health authorities as well as the Refugee Relief and Repatriation Commissioner’s (RRRC) Office which oversees the refugee relief operations in Cox’s Bazar. It played a critical role in strategically planning both the acute and protracted phase of the crisis through the 2018 and 2019 joint response plans, as well as decision making on a wide range of issues related to the general coordination of health activities. The different working groups under the health sector were formalised by the HSSAG and empowered to take technical leadership roles in their respective thematic areas, and to input into the HSSAG accordingly.

At the start of the crisis, as many health partner agencies sought to establish health facilities, standardization and distribution of health services was a challenge. One key priority was to rapidly develop minimum standards for health facilities in the refugee camps to provide a benchmark against which the health sector could monitor health facilities and to institute a basic level of equity in service provision. This was done by defining two levels of primary health care: health posts (broadly equivalent to government community clinics and counted as a basic health unit according to sphere standards) and primary health centres (broadly equivalent to government union-level sub-centres and counted as a ‘health centre’ according to sphere standards) [6]. Minimum standards for each level were developed by the HSSAG members based on national and global standards including the Bangladesh ‘Essential Health Service Package’ [7], sphere standards [6] and UNFPA’s Minimum Initial Service Package (MISP) for Reproductive Health [8]. With the endorsement from the government health authorities, these were published and shared with all health partner agencies as early as November 2017. The standards were reviewed and revised again by the HSSAG in November 2018 based on lessons learned from the previous year and shifting priorities. The target number of health posts and primary health centres relative to population size were slightly decreased, given the anticipated reduced funding and the agreed need for quality over quantity of health facilities.

Despite the existence of minimum standards, variations in quality remained a constant challenge in this response. Duplication of services were common in camps with easy road access, with shortages in camps that were more difficult to access, resulting in unequal distribution of services. A gap analysis in early 2019 identified a deficit of 9 Primary Health Centres (PHCs) and a surplus of 84 health posts in 22 priority camps. To help overcome the unequal distribution of health facilities in the camps, the health sector initiated a systematic “rationalization” exercise for primary health facilities whereby all health facilities in the above-mentioned 22 ‘priority camps’ were assessed by an inter-agency task team which reported to the HSSAG. Using a structured questionnaire, each health facility was objectively scored and a camp wise- review was done to determine which facilities should be decommissioned, relocated or kept in each camp. In total, 167 facilities were assessed as part of this exercise, of which 67 were suggested to be decommissioned on the basis of their poor quality of services and/or being located adjacent to a better performing health facility. Within 6 months, 38 (57%) of these had closed down, with the support of the government authorities whose strong involvement in every step of the rationalisation process was essential.

**Fig 1:**
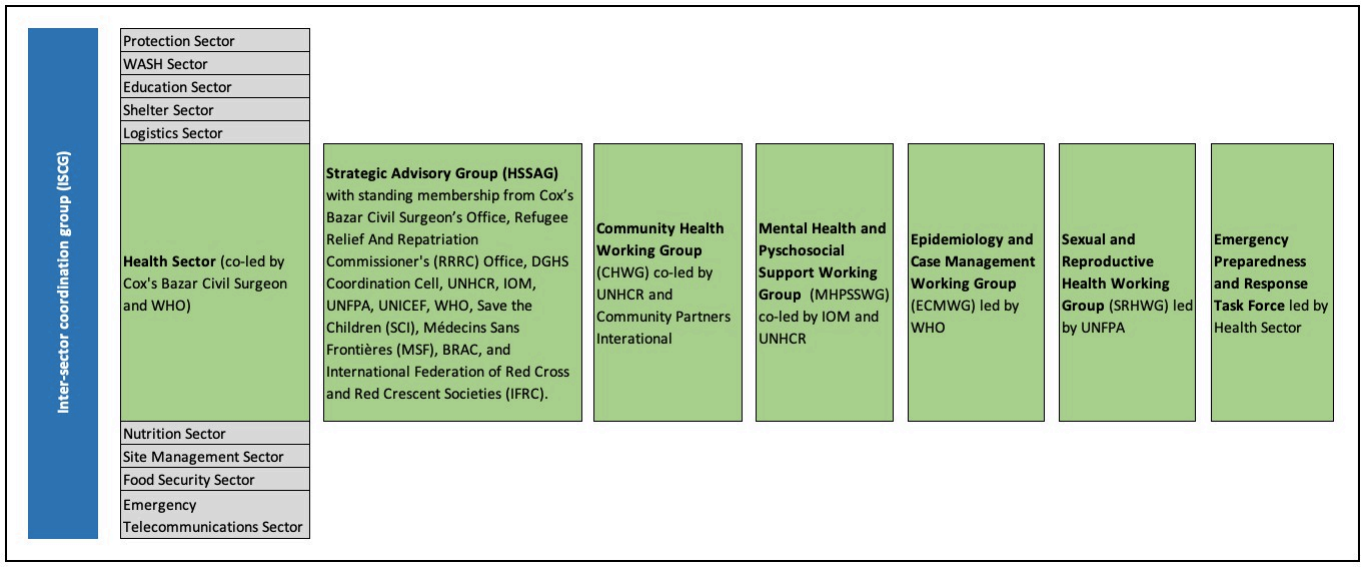
Coordination structure of humanitarian stakeholders with details of health sector coordination structure.

Until early 2018, the health sector included just two specialized working groups: Mental Health and Psychosocial Support (MHPSS) and Sexual and Reproductive Health (SRH), typical of many “health clusters”. Other groups emerged on an ad-hoc basis for example in response to the diphtheria outbreak (December 2017), to develop a joint plan for acute watery diarrhoea with the WASH sector (January 2018), and to address community health issues (March 2018). However, at the end of 2018, HSSAG reviewed the coordination structure and consolidated four working groups in which health partner agencies could be represented and jointly develop strategies and implement operations: MHPSS; SRH; community health (CH); and epidemiology and case management (ECM). In addition, a cross-cutting health sector emergency preparedness and response (EPR) taskforce was established to prepare for climatologic disaster events such as heavy monsoons and cyclones. These working groups remained very active thereafter, with updated terms of reference and selected or elected chairs from health partner agencies. Working group chairs were standing members of the HSSAG (see Fig 1).

Other accomplishments of the HHSAG have included developing a draft HIV framework for the refugee setting, developing solutions to challenges related to medical emergency referrals, and developing technical guidance and concept notes on different subjects such as the role of traditional birth attendants and the maternal death audit process in the refugee context. All HSSAG achievements can be attributed to the shared commitment of its member agencies to a fair, horizontal, strategic, unbiased and impartial approach to achieving collective health outcomes for the population in need.

Throughout, the HSSAG has relied on technical support of its working groups but remained a decision-making body first and foremost, which was essential to its success. Meanwhile, appropriate representation by health stakeholders and a strong commitment to transparency towards government authorities, health partner agencies and the intersectoral coordination group were critical in ensuring its legitimacy and acceptability.

## Epidemiology and case management

Situated in a tropical belt with a climate characterized by a distinct monsoon season between June to October, Bangladesh is particularly vulnerable to outbreaks of infectious, waterborne and other types of diseases. Increases in water-borne diseases such as diarrhoea, dysentery and cholera are usually observed during the summer months due to the combination of higher temperatures and potential flooding and or water logging from the monsoon season. Trends in vector-borne disease, such as dengue and malaria, to some extent follow the monsoon seasonality whereby increased precipitation and higher temperatures promote the proliferation of vectors thereby increasing the risk of disease transmission [9].

**Fig 2:**
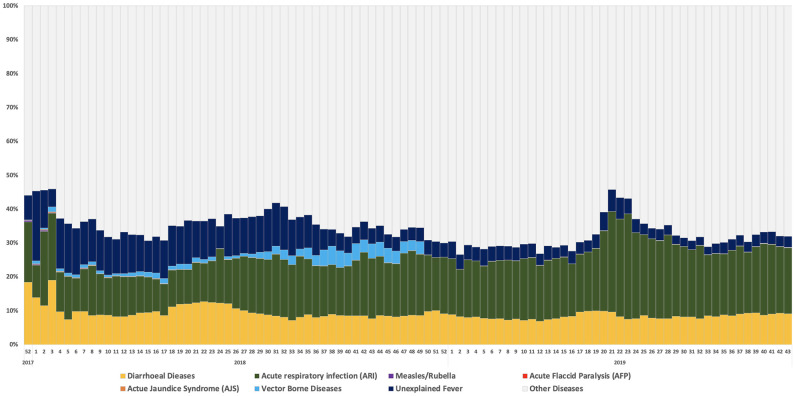
Cumulative trend in proportional morbidity for key diseases under EWARS surveillance from 1 January 2018–27 October 2019 [11].

Monitoring and preventing communicable diseases and responding to outbreaks has remained high on the priority agenda since the start of the crisis, given the crowded and unsanitary living conditions in the camps and the known lack of immunization among refugees prior to their arrival in Bangladesh. This has been the primary function of the epidemiology and case management working group (led by WHO) in support of the health sector. An infectious disease surveillance system covering the refugee camps was urgently needed at the start of the crisis, and in August 2017 WHO’s early warning alert and response system (EWARS) was implemented (initially in paper-based form and then upgraded to an electronic system from January 2018), covering 20 syndromes [10]. As of end October 2019, 86% of all health facilities were registered to report in EWARS with a cumulative reporting completeness of 89% in 2019. The proportional morbidity of diseases under surveillance in EWARS is indicated in Fig 2. Since the start of the crisis, several disease events required outbreak response activities in the refugee camps, notably outbreaks of diphtheria (2017–2018), measles (2017) and varicella (2018). This article cannot do justice to the complexities involved in all these response activities, some of which are documented elsewhere. It does, however, reflect on some of the unique characteristics in the AWD preparedness and response activities and the varicella response which to date have not been documented and which illustrate the importance of inter-sectoral collaborations in health promotion efforts.

Water, sanitation and hygiene (WASH) provisions in the camps have been constant concerns, requiring coordination between the health and WASH sectors, particularly during rainy seasons. Preparedness planning for acute watery diarrhoea (AWD) has remained a re-occurring theme throughout this response as AWD has the second highest proportional morbidity of all syndromes under surveillance, among the refugee population (see Fig 2) [11]. As early as November 2017, the first AWD preparedness plan was developed jointly with the WASH sector. The initial draft outlined the processes for disease alert investigation, the coordination mechanisms and the respective health and WASH actors’ responses to different scenarios.

**Fig 3:**
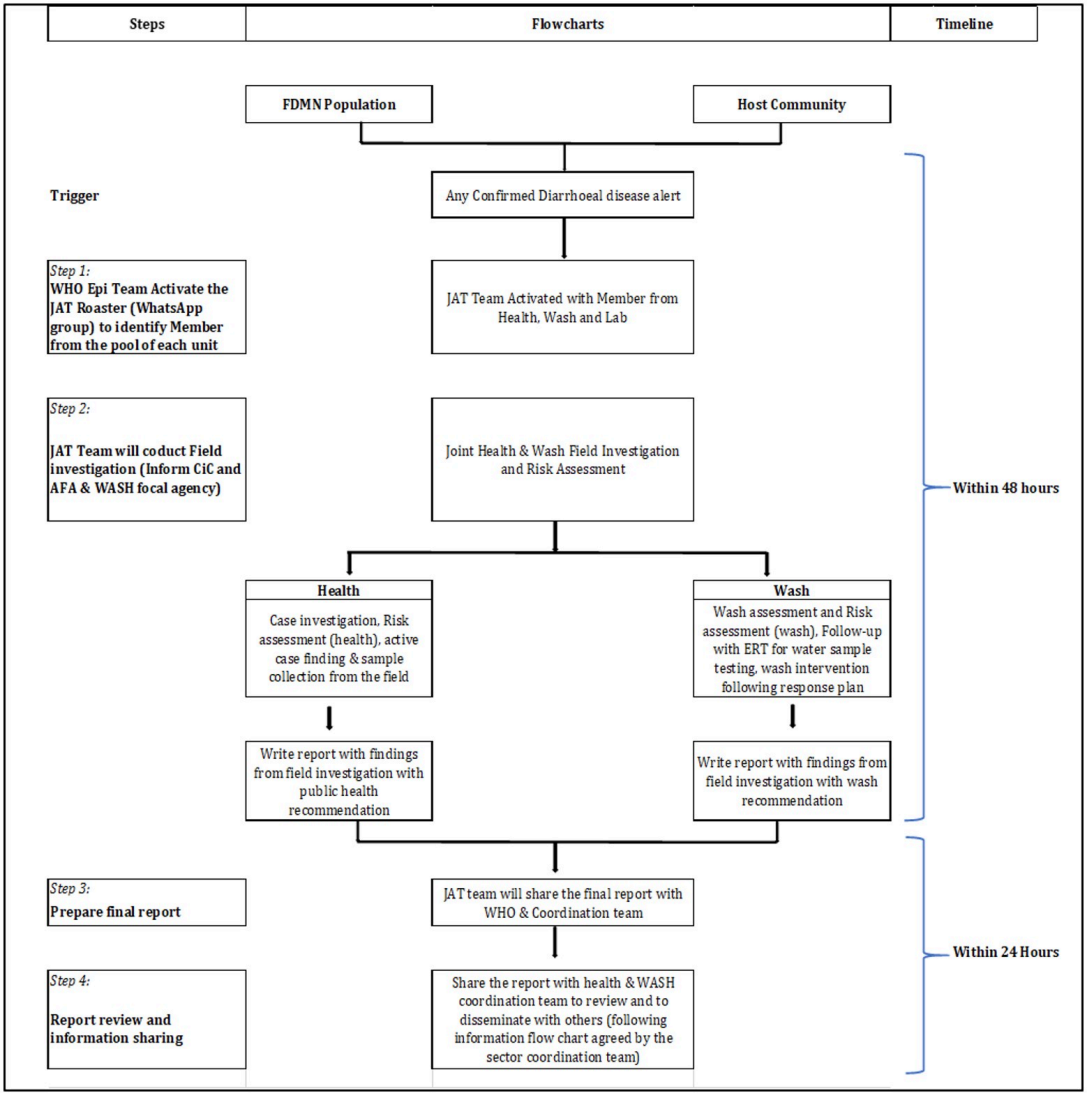
Joint assessment team operational flow-chart.

In 2019, a new response strategy of ‘joint assessment teams’ (JAT) was conceived and introduced, to investigate AWD alerts [12]. These JATs constituted trained personnel from the health and WASH sectors’ partner agencies that, under the coordination of WHO, mobilized to voluntarily conduct joint case investigation, response and follow-up activities at the field level for confirmed AWD alerts, as indicated in Fig 3. Through this mechanism, all confirmed diarrheal disease alerts were successfully investigated and timely contained in 2019, involving a joint WASH and health risk assessment (using standardized joint assessment tools), case investigation and active case search including stool specimen collection by the health actors as well as water quality testing and hygiene promotion activities by the wash actors. Importantly, JATs reported to the camp authorities as a joint WASH and health team which improved inter-sectoral coordination at the field level. This collaborative approach also helped address resource limitations, ensuring sufficient availability of staff to investigate all alerts and avoiding over-reliance on single health partner agencies. However, monitoring the implementation of recommendations remained challenging, in the absence of any direct accountability of camp-level actors towards the health and WASH sectors.

In December 2018, a varicella outbreak occurred in the camps and spread very rapidly due to close living arrangements, reaching a total of 82, 297 cases (from week 45 2018 to week 22 2019) with 10,133 cases at its peak (week 9, 2019) [11]. While the disease is self-limiting with limited risks of complications, the situation presented a challenge in that there were no available national guidelines or reference documents available. Clinical management and reporting guidelines were rapidly developed under the leadership of the epidemiology and case management working group and in collaboration with government health authorities, and partner agencies rapidly mobilised to train health care workers to ensure differential diagnosis with measles (a notifiable disease), which was essential to avoid disruptions to the measles surveillance program. One particular success of this response was the close collaboration with the education sector, to disseminate key messages and risk communication materials to the learning centres for early detection and containment of cases. More generally, the outbreak illustrated the need to be prepareded for unanticipated health events in the camps, and the importance of effective collaborations to enable rapid response when these arise.

## Sexual and reproductive health

An estimated 52% of Rohingya refugees are women and girls [13] and approximately 24.5% are women of reproductive age (WRA) aged 15–49 years [14]. Using UNFPA’s minimum initial service package calculator tool, which provides indicative estimates of maternal and newborn health, contraceptives, sexual violence, HIV, and other STIs indicators based on inputted data on number of affected populations [15], the proportion of pregnant women is estimated to be 2.4% of the total refugee population. The scale of the crisis, combined with the already over-burdened secondary health care services in Bangladesh, compounds the risk of poor health outcomes for refugee WRA, whose rights to sexual and reproductive health should continue during their displacement, as their needs do [16]. The sexual and reproductive health working group (led by UNFPA) has supported the health sector in the effort to ensure comprehensive SRH service availability and access through advocacy, innovations, collaborations and prioritisation of resources. However, two years into the crisis, many challenges persist.

The estimated maternal mortality ratio of 179/100,000 [17] is comparable to the Bangladeshi national average of 173/100,000 [18]. Ensuring that deliveries occur in health facilities and not at home is a well-recognized approach to reducing maternal and neonatal deaths [19]. At the onset of the influx, just 22% of deliveries were estimated to have occurred in health facilities [20]. This increased to 35% by June 2019 based on data collected from community health workers [21]. This increase is attributed to improved availability of ambulance-equipped 24/7 primary health facilities (from 17 in January 2019 to 32 by October 2019); establishment of referral hubs with community transport links from household level to health centres; capacity building of health care workers and community health workers; investments in procuring essential SRH commodities; and community sensitization and mobilization.

However, among the Rohingya refugees, the socio-cultural preference or expectation is to deliver at home with the assistance of traditional birth attendants, worsened by misconceptions and fears about health facilities [22,23]. Majority of deliveries still occur at home and late referrals of complicated cases are observed, both of which increase the likelihood of maternal death. To further improve the facility-based delivery rate among the refugees, supply and demand-side interventions are still needed to address the three important delays to accessing effective maternity care in a timely manner: delays in seeking, reaching, and receiving quality obstetric services [24,25]. This requires addressing the social beliefs and fears around facility-based deliveries and engaging men and other family members in health education.

Unsafe abortion is another leading cause of maternal mortality globally [26], and ensuring access to safe abortion and post abortion services is particularly important in humanitarian crisis settings where women are more vulnerable to sexual violence, unintended pregnancy and pregnancy-related complications [27]. This has been a considerable area of success in this response, as menstrual regulation (MR) and post abortion care services were introduced in 8 facilities to serve the needs of the refugees within a month of their arrival in the camps. Within two years, this increased to 37 health facilities, with more than 8000 Rohingya refugee women and girls having received abortion care. This experience not only illustrates the demand for these services, despite initial concerns to the contrary, but also demonstrates that with advocacy and a willingness to adapt, such services can be made available even in complex humanitarian settings [28].

Ensuring quality of both primary and secondary maternity services remains another persistent challenge in the SRH response. At primary health levels, there is need to scale up the quality of midwifery led care across the camps by providing mentorship support to the midwives, many of which are newly graduated and have limited professional experience. Meanwhile, ensuring access to emergency obstetric care has been a constant concern. Excluding government hospitals, which are already overburdened, the ratio of hospitals to refugees exceeds sphere standards at approximately 1 per 300,000 (sphere standard indicates at least 1 health facility offering comprehensive obstetric and neonatal care (CEmONC) per 500,000) [6]. However, these figures mask many operational challenges as the majority of the field hospitals lack continuity in sufficient qualified surgeons and anaesthesiologist to run these hospitals 24/7, which affects the reliability of their services. To help address this, a ‘hospital rotation plan’ was implemented to ensure at least one facility is available/open each day of week for emergency referrals from the camps. While this rotation plan has met with some success, it remains limited by reliability of services; blood availability; capacity levels of hospital staff and high rates of staff turnover. As a result, many emergency obstetric cases are ultimately referred to the government hospitals, often with significant delays.

In line with the MISP guidance, family planning was prioritized from the beginning of the crisis [29]. Collaborative agreements in the health facilities, allowing different health partner agencies to provide different services in the same facility, was an important modality which enabled specialized health partner agencies to work in their areas of expertise to rapidly increase coverage of family planning services [29]. In early 2018, the contraceptive prevalence rate was estimated at 33.7%, considered high for emergency settings, with injectable (70.5%) and oral contraceptive pills (28.9%) as the two most commonly used methods [30]. Until May 2018, family planning services providers in the response operated within a restrictive government policy, whereby Rohingya could only access short-term contraceptive methods due to a requirement of having a permanent address to receive long-acting reversible contraceptives (LARC). However, as a result of successful advocacy by the SRHWG and its member partner agencies, an agreement was signed in April 2018 between UNFPA and the Bangladesh Family Planning Directorate allowing UNFPA to procure and provide LARCs to Rohingya refugee women. An exception was also made to allow trained midwives to provide IUDs, although implants can still only be provided by a medical doctor [31]. Despite the high uptake of family planning and the improved availability of different methods in the refugee camps, adolescent girls in particular continue to face high barriers to accessing those services [29].

Response to sexual violence is a priority in emergencies as stipulated in the MISP which requires bridging gaps between clinical gender-based violence (GBV) services (which typically falls under the remit of health sector) and other case management support (which typically falls under the remit of GBV sub-sector under the protection sector). To ensure a coordinated response, a GBV health facility monitoring tool was developed by the health sector and supportive supervisions were jointly conducted by the health sector and the GBV sub-sector in May 2019. Based on the findings and identified gaps, facility-wise improvement plans were developed and shared with respective agencies for improvement along with technical support. This exercise also informed the development of a health sector GBV action plan to help increase availability of CMR services in the camps 24/7. This initiative would not have happened without the inter-sectoral collaboration.

Decentralization of HIV prevention and response services from the district facility to the facilities in the camps was another identified priority later in the response. It took time to address this issue, but through advocacy, by mid-2019, permissions were obtained for provider-initiated HIV testing and counselling services to be made available in selected health facilities in the camps, which was an important first step in bringing selected HIV services closer to the refugees.

## Community health

Involving members of the Rohingya population in delivery community health services, through establishing a network of community health workers/volunteers, was an increasingly important feature of this health response. The role of CHW/Vs in this crisis is particularly important given the linguistic complexities of the refugee camps, where the majority of refugees speak either Rohingya language or Burmese, in contrast to aid workers who typically speak Bangla, English or the Chittagonian dialect which resembles Rohingya but is different [32].

More than 21 health partner agencies implement community health activities through Rohingya community health workers/ volunteers (CHW/V) and one of the first priorities for the community health working group (led by UNHCR and Community Partners International) was to standardize the activities and capacity of this important cadre of the health workforce. By October 2018, a guidance note had been published by the CHWG, defining the tasks expected of the CHW/Vs both during their routine activities and during monsoon or cyclone-related event, as well as the expected profile/qualifications of CHW/Vs and required equipment. The guidance note set a target for each refugee household to be visited by a CHW/V at least once in every two-week period, with a target household coverage per CHW/V of 150–175 households per CHW/V, in line with the Sphere standard of achieving 1–2 CHW/V per 1,000 people [6].

Establishing an agreed ratio of households per CHW/V enabled the CHWG to map partner agencies’ activities and conduct a first gap analysis in October 2018. As with primary health facility coverage, overlapping CHW/V coverage was seen in many camps while gaps in service provision remained in others. A step-wise consensus building approach was used at the camp level to define and assign work coverage areas for each partner agency and, once agreed, these were mapped and published for collective understanding and reference. However, some partner agencies delayed relocating to their assigned areas while others initiated their activities without coordinating with the working group which resulted in new overlaps. These concerns were addressed by the time a second round of mapping was conducted in July 2019, by which time gaps or significant oversupply were observed in just 7 out of 34 camps. A total of 1,437 CHW/Vs were counted, and these are re-mapped every six months.

Ensuring basic and comparable level of training across the CHW/Vs is critical but challenging given varying levels of literacy and education. Based on the standard terms of reference, the working group developed training packages for the CHW/V including core material on SRH and first aid (mostly implemented through cascaded training of trainers’ model), in addition to materials developed in response to emerging risks such as diphtheria, chickenpox or dengue (mostly implemented through direct short trainings). A survey conducted in July 2019 demonstrated that 71% to 88% of CHW/Vs were trained on the topics for which training packages were developed, whereas less than 30% were trained on topics for which training packages are not available. This highlights the importance and value of developing standard packages, and the working group is continuously developing new material in response to emerging priorities, including on non-communicable diseases. To address literacy and language barriers of the CHW/Vs and the refugee households which they serve, supporting materials such as flipcharts and other job-aides are developed by different health partner agencies in Burmese, English and Bangla languages with a heavy use of pictorials to facilitate the CHW/Vs household visit. Collaborations with the risk communication experts has been critical in this effort.

It is well recognized that CHW/Vs can play and important and unique role in monitoring health outcomes as well as basic surveillance. To harness this, an online reporting tool (using Kobo collect [33]) was piloted from December 2018 in which partner agencies report bi-weekly on their CHW/Vs activities and key community-based data such as mortality and maternity outcomes, as well as any ‘unusual events’ noticed by the CHW/Vs. This was expanded to all partner agencies in April 2019, and as reporting rates have improved, the data can showcase the achievements of the CHW/Vs and enabled monitoring of trends on key health indicators.

CHW/V are trained to collect data at a household level on mortalities of which an estimated 66.4% (243/366) occur outside a health facility (based on EWARS mortality data from April-September 2019). Based on community-based mortality data collected in October 2019, a crude mortality rate of 0.2/1000 population/month and an under-five mortality of 0.5 /1000 population/month were estimated. Prospective mortality surveillance now allows for monthly monitoring of crude and under-five mortality rates for the first time in this response. Data from the online reporting tool also helped to highlight the potential impact of the CHW/Vs trainings on SRH.

The CHW/Vs also serve a unique function in the early detection and response to infectious disease outbreaks and have been highly responsive to emerging needs. For example, during the surge in suspected dengue cases in August 2019, 182 CHW/V supervisors were trained in dengue prevention and within one week they had trained 99.0% of the CHW/V workforce. Within two weeks of the initial training of trainers, CHW/Vs had visited 122,699 households (58.2% of all households), and within four weeks each household had been visited at least once with dengue prevention messages. However, challenges remain with regards to institutionalizing reporting of unusual events by CHW/Vs, and linking this with the disease surveillance, in part due to the need to adequately define unusual events, as well as competing activities of the CHW/Vs.

## Mental health and psychosocial support

Armed conflicts and natural disasters are associated with a wide range of social and psychological reactions that result from either pre-existing or emergency induced mental health conditions [34]. Despite popular assumptions that increased mental health needs among refugees and displaced populations are due to traumatic experiences before the displacement, it is now recognized that that daily stressors and the lack of certainty about the future also play a significant role in exacerbating the impact of these events in the long run [35,36]. A recent systematic review found that the prevalence of mental disorders in conflict affected populations is 22.1% of which 13% are mild mental disorders (mild forms of depression, anxiety and post-traumatic stress disorder), 4% are moderate disorders (moderate forms of anxiety, depression and post-traumatic stress disorder), and 5.1% are for severe disorders (severe forms of depression, anxiety and post-traumatic stress disorder, schizophrenia, and bipolar disorder) [37]. In the case of the Rohingya refugees, many experienced unprecedented trauma in Myanmar and during their journey to Bangladesh, while environmental daily stressors are found to compound the relationship between the exposure to trauma and the distress symptoms they experience [38].

Many challenges impede effective provision of mental health and psychosocial support services for the Rohingya refugees, including risks associated with monsoon and cyclone seasons. Heavy rainfall and landslides can lead to loss of lives, loss of property, service interruption and social disconnection, which can exacerbate mental health conditions. Another major challenge is the language barriers between the Rohingya communities and local aid workers [39]. There is also a lack of pre-existing information about mental health and the related cultural considerations of the Rohingya refugees [40]. Such cultural factors and barriers affect how individuals and communities perceive mental health issues and mental health services, and consequently impact the help seeking behaviour and the service utilization by target communities. Regarding human resources, significant gaps exist in specialized mental health services as there is only one psychiatrist who works in Cox’s Bazar district hospital and there is no psychiatric ward. Many of the psychologists working in the humanitarian response are recent graduates with limited clinical training or training on psychotherapeutic approaches.

The Mental health and psychosocial support working group (led by IOM and UNHCR), has supported the health sector in coordinating specialized MHPSS partner agencies. Strategies were adopted to address the highlighted challenges including community based mental health and psychosocial approaches aimed at restoring the pre-existing social fabric and promote community resilience by using existing strengths and resources within the affected populations. Recruitment of community psychosocial volunteers and lay counsellors from the Rohingya community has been a successful approach in this response to bridging the communication and cultural gaps between the humanitarian community and the refugee communities. These volunteers facilitate both group and individual activities e.g. peer support groups, community psychoeducational workshops and individual psychosocial sessions when indicated. Involvement of traditional healers and religious leaders in planning, implementation and evaluation of different aid initiatives have also helped to inform the services’ design by the community perceptions and expectations. Integration of mental health into primary health care services has been identified as an important strategy to help bridge the gap in specialized mental health services and to improve the acceptability, access and sustainability of services together with decrease in the stigma associated with mental illness and mental health services [41–43]. Medical doctors, nurses, and medical assistants were enrolled in capacity building programs that include on job support and supervision by psychiatrists recruited by partner agencies and by October 2019, 81% (26/32) of primary health centres had at least one healthcare worker trained on MhGAP according to monitoring data from the health sector.

## Emergency preparedness and response

Bangladesh’s geographical location makes it particularly vulnerable to cyclones and seismic risk [5]. Its climate is tropical characterized by mild winters (October to March), and hot, humid summers (March to June). A warm and humid monsoon season lasts between June and October, supplying most of the country’s annual rainfall with two cyclone seasons documented before and after the monsoon season [44,45]. Several major natural hazards for the Rohingya refugee camps were characterised by the natural hazards task force under the coordination of the Inter Sectoral Coordination Group [46], with a “high risk” of cyclone impact determined within Cox’s Bazar district including areas where the refugee camps are located [47]. Tropical cyclones can carry heavier rainfall compounding the existing risks from the monsoon season such as floods and landslides. Storm surge is a secondary potential hazard arising from an approaching cyclone [45,48,49] which can reportedly reach up to 8 metres during severe cyclonic winds [50].

From August 2017 to October 2019, no major cyclones or severe natural hazards have affected Cox’s Bazar district of Bangladesh. However, recognising these seasonal risks and their unique potential to create a disastrous ‘emergency within an existing emergency’ for the refugees and surrounding host communities, all sectors are expected to integrate emergency preparedness and response readiness into their respective plans. Accordingly, the health sector integrated the health emergency preparedness and response readiness process into its overall coordination mechanism from the start, linking with humanitarian agencies, the Bangladeshi Military and government actors. For health, a severe cyclone could have direct public health consequences (injuries, drownings, and increased waterborne diseases) and indirect consequences (further reduced access to health services due health facility damage and impaired movement; and risk of epidemics). Through consultation with the technical working groups, plans were made to minimize the impact of the potential natural hazards on the health system and health care delivery. These were incorporated into a single health sector contingency plan which describes the preparedness and response activities for the cyclone and monsoon seasons according to levels of severity.

Recognising that health actors may not be the first on the ground, the overall preparedness approach across the different sectors was to strengthen the community’s capacity to provide cyclone early warning messages (using a flag-based system which is implemented across Bangladesh), and to act as first responders. In line with this, the community health volunteers were trained on first aid and equipped with first aid kits. In addition, hospitals’ availability and readiness to provide trauma management were mapped, and hospitals developed their respective contingency plans. Overall, the health sector contingency plan aimed to resume critical health services as soon as possible after an event, through medical stockpiling and trained mobile medical teams which can be deployed in case of severe damage to health infrastructure and which will be granted special access. A dispatch and referral hotline was established since 2018 for requesting emergency ambulance support and mobile medical teams.

However, several challenges constrain the contingency planning efforts, many of which fall outside the scope of the health sector but impact the health response. First, there is no overall plan to evacuate the Rohingya refugees to cyclone shelters in case of a cyclone and there are insufficient cyclone shelters to cover the host communities. In March 2018, it was estimated that existing shelters cover approximately 20% of the population in the sub-districts where the refugees are located [50]. Tele-communication is another important challenge, due to restrictions on owning high frequency radios and the possibility of communication black-out in the event of a severe cyclone. Such a scenario would severely hinder ambulance dispatches and mass casualty management and requires prepositioning of ambulances and medical teams during the early warning phase of a cyclone. Road access is also problematic in the area, with only one main access road to the main refugee camps which could foreseeably be cut off by floods or other obstructions. Given the potential impact on health service delivery, the health sector was vocal in advocating for improvements in these areas, and played a prominent role in the inter-sectoral coordination group’s emergency preparedness working group. However, as of October 2019, these concerns continued to threaten the effectiveness of all sectors’ emergency planning efforts particularly relating to potential high-impact weather emergency such as cyclones.

## Conclusion

Overall, while numerous gaps and challenges remain to be addressed, this paper has highlighted several of the health sector’s accomplishments over the past two years. These achievements, which occurred in a challenging context with a plethora of health partner agencies, can largely be attributed to the strong collaboration and partnership that was fostered from the outset of the crisis and which has continued throughout. WHO is mandated at the global level by the humanitarian cluster coordination structure to lead the sector (or cluster) response to health emergencies. While there is a clear need for this leadership role, the experience from this Rohingya refugee response has demonstrated the real benefits of fostering a “collective coordination” approach.

The creation of an inter-agency HSSAG within two months of the crisis onset, with a mandate for decision-making and strong representation from both health partner agencies and the different government authorities, was critical. It created a participatory rather than a top-down coordination structure which was more acceptable to the wider network of health partner agencies and introduced a ‘shared accountability’ to the strategic decisions and their outcomes. In addition, delegating the leadership of technical working group coordination to specialised health partner agencies with defined terms of reference enabled the health sector to draw on these respective organisations’ technical expertise, while empowering the working groups to develop novel initiatives, solutions and ideas in consultation with their partner agency members, for wider review and endorsement by the HSSAG.

Despite the unique and changing challenges of operating in this crisis, this coordination model proved effective and adaptive, and lessons learned can hopefully be beneficial to other crises and contexts. The leadership, collaborations and partnerships nurtured over the past two years remain as important going forward, as the attention and funding moves away from Bangladesh to other more acute crises globally, threatening the high standards set by the health sector.
